# Combination Pharmacology for ALS: A Mechanistic Rationale

**DOI:** 10.3390/ijms27167404

**Published:** 2026-08-19

**Authors:** Jeffrey Rosenfeld, Shiran Salomon-Zimri, Ferenc Tracik

**Affiliations:** 1 Center for Restorative Neurology, Department of Neurology, Loma Linda University Health, Loma Linda, CA 92354, USA; 2 NeuroSense Therapeutics, Herzliya 4672562, Israel; shiran@neurosense-tx.com (S.S.-Z.); f.tracik@gmx.de (F.T.)

**Keywords:** combination therapy, ALS, neurodegeneration, clinical trials, mechanistic rationale

## Abstract

Amyotrophic lateral sclerosis (ALS) involves multiple converging pathogenic mechanisms, including glutamate excitotoxicity, oxidative and endoplasmic-reticulum stress, mitochondrial dysfunction, neuroinflammation, iron dysregulation, and altered microRNA processing. Expecting a single pharmacologic intervention to meaningfully alter such a complex disease has proven overly optimistic and is reflected by the modest clinical benefits of approved monotherapies. This review outlines the mechanistic foundation and translational rationale for combination pharmacology in ALS. Drawing from paradigms in oncology, infectious disease, and other neurological disorders, it explores how rational multi-target strategies, whether synergistic, complementary, or pathway-divergent, may better address the multifactorial biology of ALS. The review also discusses recent mechanistic examples and design principles for advancing this therapeutic paradigm.

## 1. Introduction

Amyotrophic lateral sclerosis (ALS) is a heterogeneous neurodegenerative disorder characterized by progressive degeneration of upper and lower motor neurons. The disease encompasses a broad spectrum of mechanisms, including excitotoxicity, oxidative stress, mitochondrial dysfunction, neuroinflammation, dysregulated microRNAs, iron dysregulation, abnormal lipid metabolism, ganglioside interaction and disordered protein metabolism [1,2,3,4,5,6,7,8]. Addressing multiple pathophysiological mechanisms has been suggested to result in more effective therapeutics compared with monotherapy [9]. Clinically, ALS exhibits variability in phenotype and rate of progression, with both sporadic (approximately 90–95%) and familial (approximately 5–10%) forms.

Pharmacologic development has yielded only limited benefits, with three approved agents providing modest slowing of functional decline. The failure to stratify clinical trials by mechanism or phenotype likely dilutes treatment effects. Enrichment approaches that narrow inclusion criteria have improved signal detection, as shown in prior success with subpopulation-focused designs [10,11].

An alternative approach in investigations for new pharmacologic therapies has been to combine drugs with different potentially therapeutic targets, expanding the potential for a treatment effect. If, for example, a pathophysiologic mechanism was more relevant at a particular stage of motor neuron degeneration, then at any one time the population of vulnerable motor neurons may be affected by a different primary pathology, depending upon their stage of degeneration. Combining drugs would potentially expand the chance of detecting a significant treatment effect.

Monotherapy treatment trials for ALS/MND have been most common and combination therapy has not been extensively studied. In a complex neurodegenerative disorder such as ALS, this approach is both new and perhaps underappreciated as an important therapeutic strategy for new drug development. The approach, long validated in oncology, infectious disease, and epilepsy, may hold similar promise in ALS [12].

## 2. Rationale for Combination Approaches

**Figure 1:**
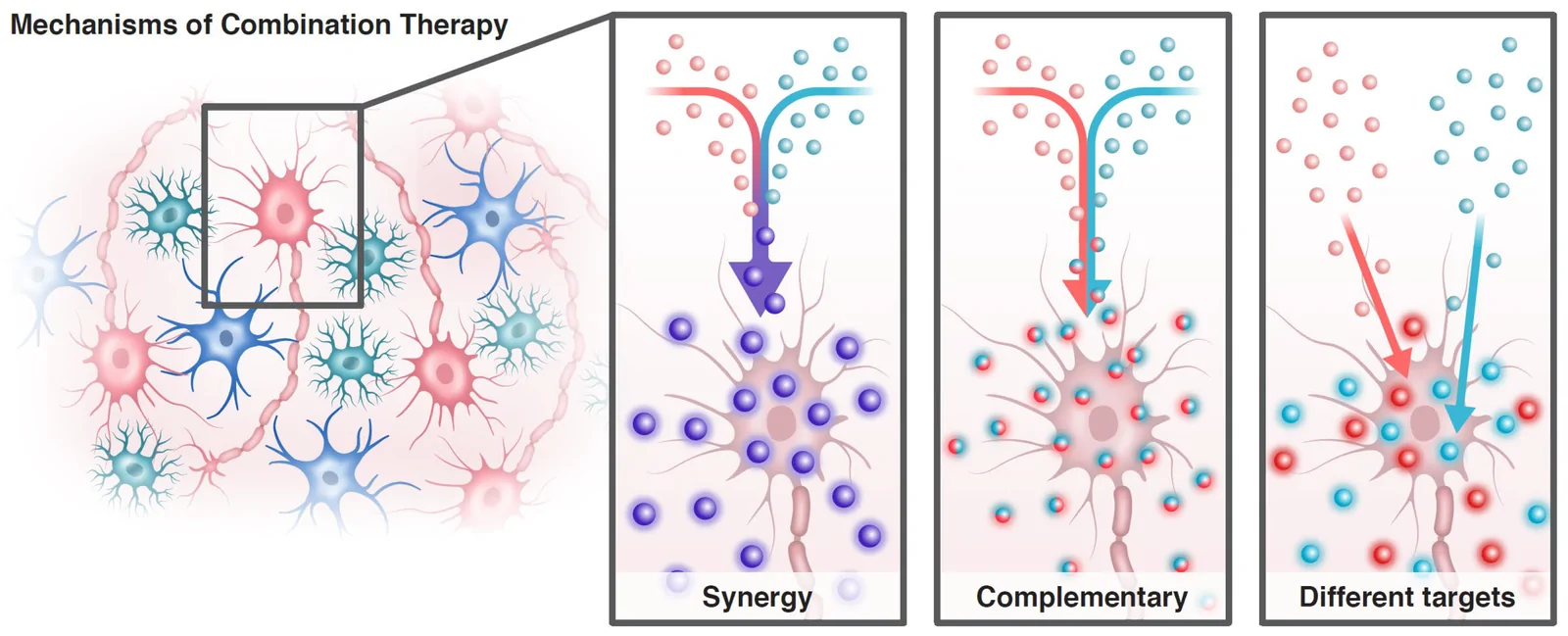
Conceptual framework for combination pharmacology in ALS illustrating synergistic, complementary, and multi-pathway strategies targeting neurodegenerative mechanisms. The two colored arrows are meant to depict two different drugs in a combination therapy. In Synergy, the two colored arrows combine to make a new color (purple). In the Complementary and Multi-target examples the two drugs (colors) have different sites of action with different implications.

Combination therapy can enhance efficacy through several mechanistic frameworks (Figure 1). First, synergy may arise when two agents act on related molecular pathways to produce a greater combined effect than alone. Second, complementary pharmacokinetics or pharmacodynamics may occur when one drug enhances another’s exposure, duration, or target engagement. Third, multiple-pathway coverage allows independent mechanisms to be modulated concurrently, reflecting ALS’s complexity [13]. Multiple examples exist, in a wide variety of indications, where combination therapy has surpassed efficacy of monotherapy and is now standard of care.

## 3. Synergistic Drug Actions

Synergistic strategies are well established in complex diseases such as cancer and HIV, aiming to improve efficacy, reduce toxicity, and delay resistance. In ALS, an early example was the combination of riluzole and minocycline. Riluzole acts primarily through inhibition of glutamatergic transmission and sodium-channel modulation, whereas minocycline suppresses excitotoxic calcium influx. Preclinical studies demonstrated increased brain exposure to riluzole in the presence of minocycline, mediated by P-glycoprotein inhibition [14,15]. However, human trials using high-dose minocycline revealed toxicity and faster progression [16,17]. This underscores the importance of dose optimization and mechanistic understanding when developing synergistic regimens.

Comparable synergy principles underpin combination strategies in epilepsy [18,19] and Alzheimer’s disease [20,21,22,23,24,25,26,27,28,29], where multi-mechanism regimens have improved outcomes or sustained function compared with monotherapy. The NMDA receptor antagonist memantine has been combined with the cholinesterase inhibitors donepezil, galantamine, and rivastigmine for the treatment of Alzheimer’s disease. A systematic review of nine RCTs found that compared with monotherapy, combination therapy had a significant effect on cognition and clinical global impression, but not on activities of daily living or behavioral and psychological symptoms of dementia [20,21,23,24,25,27,28]. Memantine has also been evaluated in a combination with galantamine, showing significant cognitive benefit compared with galantamine alone; cognitive decline occurred after discontinuation of galantamine.

The antioxidant idebenone and the iron chelator deferiprone have been investigated in patients with Friedreich’s ataxia with reports of improvements in neurological and gait outcomes [30,31]. Synergistic combinations of gene therapy and non-biologics have also been trialed in spinal muscular atrophy. Addition of the pre-mRNA splicing modifier risdiplam or the antisense oligonucleotide nusinersen with the gene therapy onasemnogene has been reported to be efficacious in patients with spinal muscular atrophy type 1; however, large-scale clinical studies are required to determine if the combination is more effective than a single intervention [32,33].

## 4. Complementary Pharmacokinetics and Pharmacodynamics

Complementary pharmacology exploits drug–drug interactions to optimize therapeutic exposure and durability. A concomitant medication can slow the metabolism and enhance efficacy of a therapeutic drug. Many examples of this combination benefit exist.

The fixed-dose combination of dextromethorphan and quinidine for pseudobulbar affect exemplifies this concept and may have particular relevance in ALS: quinidine inhibits CYP2D6 metabolism and enhances CNS availability of dextromethorphan [34,35]. In 2010 the combination was approved by the FDA for treating pseudobulbar affect and the combination has been tested in clinical trials for AD, PD and ALS [34]. Dextromethorphan (DEX) is a low-affinity N-methyl-D-aspartate receptor (NMDA) antagonist and sigma-receptor agonist, which might also protect neurons by reducing glutamate cytotoxicity [36,37]. Quinidine elevates the bioavailability of dextromethorphan in the body by two mechanisms: lowering its metabolism by the liver and inhibiting the protein pump P-glycoprotein at the blood–brain barrier (BBB) [35]. Slowing dextromethorphan metabolism enhances the availability of the drug in the body which allows prolonged and sustained effects.

Similarly, the combination of levodopa with carbidopa and entacapone in Parkinson’s disease prolongs central dopaminergic signaling while minimizing peripheral conversion [38,39]. In ALS, applying similar principles could potentiate drugs with limited CNS penetration or short half-lives by pairing them with agents that modulate transport or metabolism [40,41,42,43,44,45,46,47,48,49]. Carbidopa prevents levodopa from being converted prematurely into dopamine in the periphery, reducing the amount of levodopa required to produce a response by about 75%, increasing the half-life of levodopa, and reducing some side effects. Entacapone can be added to this combination approach to further enhance the primary effect of L DOPA [38], increasing L-DOPA delivery to the brain and prolonging its action by increasing the drug’s half-life [39].

Drug combinations may also enable the use of lower drug doses than would be used in monotherapy. The dopamine agonist pramipexole and the MAO-B inhibitor rasagiline are used in standard treatment of Parkinson’s disease [48]. A low-dose combination of the two drugs was reported to be superior to monotherapy with either drug alone and the lower doses may mitigate some side effects typically associated with dopamine agonists [42] (see additional examples in Table 1 and Table 2).

## 5. Targeting Multiple Pathways and Emerging Combination Therapies

ALS pathogenesis arises from the convergence of multiple biological processes, including RNA dysregulation, glutamate-mediated excitotoxicity, oxidative stress, mitochondrial failure, neuroinflammation, and iron accumulation [50,51,52,53]. This multifactorial nature supports the rationale for polypharmacology, whereby multiple mechanisms are addressed simultaneously through rationally designed or fixed-dose combinations.

A notable example of this concept is the fixed-dose combination of sodium phenylbutyrate and tauroursodeoxycholic acid (PB–TUDCA), which was intended to modulate endoplasmic-reticulum stress and mitochondrial dysfunction. Although early trials suggested functional slowing, subsequent confirmatory data failed to demonstrate significant efficacy, leading to its withdrawal from the market [54,55,56,57,58,59]. This experience reinforces the importance of translational biomarker anchoring and mechanistic validation when designing combination regimens.

PrimeC, a fixed-dose combination of ciprofloxacin and celecoxib, exemplifies a next-generation multi-target approach in ALS. This regimen integrates anti-inflammatory, iron-chelating, and microRNA-modulatory mechanisms, with preclinical and clinical data demonstrating safety, target engagement, and modulation of disease-relevant biomarkers [60,61,62]. Based on results from the PARADIGM phase 2b study, a global, multicenter phase 3 trial (PARAGON) is being initiated to further evaluate PrimeC’s efficacy and confirm its disease-modifying potential.

Beyond fixed combinations, the field is witnessing the conceptual and practical emergence of biologic and small-molecule combination regimens that address convergent immune and inflammatory pathways. A combination therapy with abatacept (CTLA4-Ig) and interleukin-2 (IL-2) was investigated for safety and also the ability to suppress markers of oxidative stress, inflammation, and degeneration in ALS [63]. Similarly, pharmacologic inhibition of the NLRP3 inflammasome is being evaluated as an add-on strategy to mitigate innate immune activation and downstream neurotoxicity [64]. These examples illustrate the transition from sequential single-agent exploration to mechanism-driven, multi-agent regimens specifically designed to target intersecting pathways implicated in ALS pathogenesis.

The integration of combination therapy concepts into ALS development reflects a broader paradigm shift toward mechanistic convergence, in which rational pairing, biomarker-based validation, and enriched patient stratification are prioritized. This framework aims to capture synergistic efficacy while minimizing redundancy, thereby offering a scientifically grounded path toward more effective disease modification.

In epilepsy management, multi-drug therapy on disparate targets has been beneficial as well. In a multicenter study of 347 patients with partial or generalized seizures that were uncontrolled on sodium valproate monotherapy, the combination of lamotrigine with sodium valproate, carbamazepine, or phenytoin increased response rates to 64%, 41%, and 38%, respectively [65]. Other studies also report that AED combinations with different MOAs had the greatest effectiveness measured by persistence of effect and lower risk of hospitalization and emergency department visits [19,66]. Multi-drug therapy with three or more antiepileptics, however, was associated with higher rates of adverse events compared with monotherapy and combination of two AEDs [67].

Representative examples of multiple available therapies, in multiple conditions, are shown in Table 1 and Table 2.

## 6. Design Implications for ALS Clinical Trials

ALS trials have traditionally followed a single-agent, parallel-group design. However, adaptive, factorial, and platform frameworks now enable simultaneous evaluation of multiple combinations and dosing paradigms while limiting placebo exposure [68,69]. These designs can identify additive or synergistic effects earlier, integrate biomarker readouts such as neurofilament light chain (NfL) and microRNA panels, and refine mechanistic hypotheses.

Given our current knowledge of multiple parallel, serial or complementary mechanisms of disease pathogenesis in ALS, combination drug therapy represents not only a logical alternative approach but perhaps an explanation of why, historically, there have not been more robust outcomes from prior trials. The few negative findings from prior two-drug combination trials in ALS do not diminish the rationale for combination pharmacology but highlight the need for translational precision and biomarker-anchored design. Mechanistic enrichment and exposure–response modeling will be essential to identify clinically meaningful effects.

**Multiple potential benefits of combination (multi-drug) therapy might, therefore include:**

- Multi-pathway targeting (e.g., inflammation, iron dysregulation, endoplasmic reticulum stress or mitochondrial dysfunction).
- Potential synergy and larger effect size than single-agent trials.
- Broader therapeutic efficacy as different pathological processes might be evident concurrently accounting for different stages of motor neuron loss.

**Several pitfalls of such an approach need to also be considered:**

- Attribution, as it may be difficult to know which component drives benefit or harm.
- Complex safety and pharmacokinetics/pharmacodynamics (PK/PD) monitoring: interactions and cumulative toxicity.
- Regulatory complexity: fixed-dose cocktails vs. separate approvals; labeling and reimbursement.
- Statistical and operational burden: larger sample sizes, more endpoints, and the need for careful modeling of background therapies.

Critiques of former clinical trials and our approach(es) to clinical trial development in ALS have been well studied [70,71,72]. Often, prior failed trials are attributed to methodological concerns, lack of proper preclinical, phase 1 or phase 2 design, or perhaps absent biomarker data on drug target engagement or disease heterogeneity [70,73]. The reality, however, that optimal disease modification might necessitate targeting multiple drug targets simultaneously has not been as extensively addressed. If multiple pathogenic mechanisms were simultaneously driving disease progression, perhaps by different mechanisms affecting motor neurons in different stages of degeneration, the most optimal study design might still fail to detect clinical efficacy. Currently, our understanding of multiple pathogenic mechanisms exceeds our understanding or experience for combination, multi-drug therapy. It may require a paradigm shift in our approach to ALS therapeutic trials to reach the next generation of therapeutic advances in this complex neurodegenerative disease.

## 7. Conclusions

ALS is a complex neurodegenerative disease. Despite our understanding of a number of relevant mechanisms, current treatment options are limited to modest disease-modifying therapies. Clinical, genetic and biochemical heterogeneity may also significantly confound efforts to measure efficacy.

The concept of combining relevant therapies has become increasingly recognized and may significantly improve our ability to detect treatment effects, along with other techniques such as cohort enrichment. The strategies for combination therapy include synergistic combinations, complementary combinations or combinations targeting disparate disease processes. Future trials are greatly needed taking into consideration these mechanisms of combining drugs.

Our prior clinical trials of monotherapy have not yielded the promise predicted by the strength of the preclinical data. In retrospect it may have been too optimistic to expect more dramatic treatment effects from monotherapy when tested in a very heterogeneous condition. We hypothesize that single-drug targeted therapy may never be sufficient to address a condition like ALS with the high likelihood of multifocal intracellular pathological processes.

Many lessons can be learned from other fields of medicine such as infectious disease and oncology. As we enter a new era in the treatment of ALS, aided by multiple approved disease-modifying therapies, we are confronting an opportunity and challenge to interpret all subsequent therapies to be tested in the context of these drugs, a multi-drug therapy trial by default. Our challenge will be to expand this momentum to devise logical combination therapies addressing synergy, complementary therapies and disparate targets.

## Figures and Tables

**Table 1:** Representative examples of combination drug therapy in multiple disparate medical conditions.

Indication	Combination Regimen	Mechanistic Rationale	Key Outcomes
HIV	INSTI + 2 NRTIs	Multi-step viral life-cycle blockade	↓ AIDS progression & mortality > 70%
Tuberculosis	INH + rifampin + pyrazinamide + ethambutol	Multi-target bacterial killing; resistance suppression	WHO cure rates > 90%
*H. pylori*	PPI + bismuth + tetracycline + metronidazole	Multi-mechanism eradication + acid suppression	Eradication > 90%
Pseudobulbar affect	Dextromethorphan + quinidine	CYP2D6 inhibition ↑ DM exposure; sigma-1/NMDA modulation	Significant symptom reduction
Epilepsy	Valproate + lamotrigine	Distinct ion-channel actions → pharmacodynamic synergy	Improved seizure control
B-cell lymphoma	R-CHOP	Cytotoxic + immunologic synergy	Survival benefit vs. CHOP
Hepatitis C	Sofosbuvir + ledipasvir	Parallel inhibition of viral replication machinery	SVR 95–100%

**Table 2:** Available combination drug therapies for a variety of medical conditions.

Drug Name	Active Ingredients	Indication/Use
Depakote ER + adjuncts (regional/under study)	Valproate + adjunctive agents	Epilepsy and bipolar disorder; some regions market co-packaged combinations
Stavzor + lamotrigine (co-packaged in select markets)	Valproic acid + lamotrigine	Epilepsy; commonly co-administered, occasionally marketed together
Tarka	Trandolapril + verapamil	Hypertension; verapamil also used off-label in migraine prophylaxis
Emerging Alzheimer’s combinations (clinical trials)	GLP-1 agonists + PDE5 inhibitors	Investigational regimens aimed at slowing cognitive decline
Xadago (co-administered with levodopa)	Safinamide + levodopa	Adjunct therapy for Parkinson’s disease
Nuedexta	Dextromethorphan + quinidine	Pseudobulbar affect (PBA)
Sinemet	Carbidopa + levodopa	Parkinson’s disease
Stalevo	Carbidopa + levodopa + entacapone	Parkinson’s disease; extends levodopa effect via COMT inhibition
Duopa	Carbidopa + levodopa (intestinal gel)	Advanced Parkinson’s disease requiring continuous jejunal infusion
Rytary	Carbidopa + levodopa (extended-release)	Parkinson’s disease; smoother pharmacokinetic profile
Namzaric	Memantine + donepezil	Alzheimer’s disease
Treximet	Sumatriptan + naproxen	Acute migraine
Midrin (legacy product)	Isometheptene + dichloralphenazone + acetaminophen	Migraine and tension headaches (less commonly used today)
Qsymia	Phentermine + topiramate	Obesity; topiramate also an antiepileptic

## Data Availability

No new data were created or analyzed in this study. Data sharing is not applicable to this article.
